# High-density genetic map construction and QTLs identification for plant height in white jute (*Corchorus capsularis* L.) using specific locus amplified fragment (SLAF) sequencing

**DOI:** 10.1186/s12864-017-3712-8

**Published:** 2017-05-08

**Authors:** Aifen Tao, Long Huang, Guifen Wu, Reza Keshavarz Afshar, Jianmin Qi, Jiantang Xu, Pingping Fang, Lihui Lin, Liwu Zhang, Peiqing Lin

**Affiliations:** 1 Key Laboratory of Ministry of Education for Genetics, Breeding and Multiple Utilization of Crops; Key Laboratory of Crops by Design, Fujian Agriculture and Forestry University, Fuzhou, 350028 People’s Republic of China; 2 Biomarker Technologies Corporation, 101300 Beijing, China; 3 Guangxi University, 530000 Nanning, China; 4 Eastern Agricultural Research Center, Montana State University, 59270, Sidney, Montana USA

**Keywords:** *Corchorus capsularis* L, SLAF, Genetic map, QTL, Plant height

## Abstract

**Background:**

Genetic mapping and quantitative trait locus (QTL) detection are powerful methodologies in plant improvement and breeding. White jute (*Corchorus capsularis* L.) is an important industrial raw material fiber crop because of its elite characteristics. However, construction of a high-density genetic map and identification of QTLs has been limited in white jute due to a lack of sufficient molecular markers. The specific locus amplified fragment sequencing (SLAF-seq) strategy combines locus-specific amplification and high-throughput sequencing to carry out *de novo* single nuclear polymorphism (SNP) discovery and large-scale genotyping. In this study, SLAF-seq was employed to obtain sufficient markers to construct a high-density genetic map for white jute. Moreover, with the development of abundant markers, genetic dissection of fiber yield traits such as plant height was also possible. Here, we present QTLs associated with plant height that were identified using our newly constructed genetic linkage groups.

**Results:**

An F_8_ population consisting of 100 lines was developed. In total, 69,446 high-quality SLAFs were detected of which 5,074 SLAFs were polymorphic; 913 polymorphic markers were used for the construction of a genetic map. The average coverage for each SLAF marker was 43-fold in the parents, and 9.8-fold in each F_8_ individual. A linkage map was constructed that contained 913 SLAFs on 11 linkage groups (LGs) covering 1621.4 cM with an average density of 1.61 cM per locus. Among the 11 LGs, LG1 was the largest with 210 markers, a length of 406.34 cM, and an average distance of 1.93 cM between adjacent markers. LG11 was the smallest with only 25 markers, a length of 29.66 cM, and an average distance of 1.19 cM between adjacent markers. ‘SNP_only’ markers accounted for 85.54% and were the predominant markers on the map. QTL mapping based on the F_8_ phenotypes detected 11 plant height QTLs including one major effect QTL across two cultivation locations, with each QTL accounting for 4.14–15.63% of the phenotypic variance.

**Conclusions:**

To our knowledge, the linkage map constructed here is the densest one available to date for white jute. This analysis also identified the first QTL in white jute. The results will provide an important platform for gene/QTL mapping, sequence assembly, genome comparisons, and marker-assisted selection breeding for white jute.

## Background

Jute (*Corchorus* sp.) is the second most cultivated fiber crop globally after cotton, and is extensively grown in India, Bangladesh, China, Thailand, Myanmar, and Nepal [1]. White jute (C. *capsularis*) and dark jute (C. *olitorius*) are two most popular cultivated *Corchorus* species. Both species have 2n = 2× = 14 chromosomes [2]. Jute is of importance to textile and paper industries as it is a valuable ingredient for producing paper and fine textiles, as well as being a renewable source for biofuel [3]. Jute fibers exhibit a characteristically high luster, good moisture absorption performance, rapid water loss capacity, and easy degradation [4]. Therefore, the use of jute has received considerable attention in many countries [5]. However, compared with other related bast fiber crops, there has relatively little progress in genetic improvement in jute in recent times, and no new breeding approaches have been developed over the past seven decades [6]. The advances in sequencing technologies have offered an alternative approach to traditional breeding methods and jute breeders and biologists are now giving more attention to the use of molecular tools to increase fiber quality and yield, and to improve agronomic traits [6].

A genetic map, especially a high-density genetic map, provides an important foundation for mapping quantitative trait loci (QTLs) [7–10] and anchoring sequence scaffolds [11–13]. High-density genetic maps have been used to reveal genome composition and for selection of high throughput superior traits in many species [14]. Previous research in jute has shown that plant height is a major component of fiber yield, and is strongly correlated with both fiber content and fiber yield [15]. Thus, construction of a high quality genetic map and identification of QTLs for plant height are necessary for further development of white jute.

Genetic maps have been developed in dark jute and used for identification of QTLs. For example, Kundu et al. developed a linkage map containing 503 restriction site associated DNA (RAD) markers spanning 358.5 cM, and 9 QTLs for histological fiber content were detected [15]. Topdar et al. reported a microsatellite genetic map and identified 26 QTLs for fiber quality, yield, and yield-related traits [16]. Sultana et al. [17], constructed a linkage map with three linkage groups covering 87.3 cM using 10 inter-simple sequence repeats (ISSR) in an F_2_ population from a cross between two dark jute genotypes. Haque et al. reported a linkage map including 40 randomly amplified polymorphic DNA (RAPD) markers [18]. Chen et al. developed a genetic linkage map using 122 sequence-related amplified polymorphism (SRAP) loci and three morphological markers, with an average marker interval of 17.86 cM [19]. Das et al. constructed a linkage map covering 784.3 cM using 36 polymorphic simple sequence repeats (SSRs) markers in a recombinant inbred line population from a cross between two dark jute genotypes, and 21 QTLs were identified for eight fiber yield traits and for a fiber quality trait (fiber fineness) [20].

However, genetic map and QTL information for white jute (C. *capsularis*) is much more limited. Chen et al. reported a linkage map comprising 119 markers that covered 2185.7 cM with a mean density of 18.7 cM per locus [2]. Recently, nine linkage groups were identified in a genetic map with an estimated length of 2016 cM and average marker interval of 4.2 cM; this map included a final set of 458 markers (48 SSRs and 410 SNPs) [21].

As described above, genetic maps for white and dark jute exist; however, the total number of markers on the LGs of most of these maps is limited and some of the mapped markers have no sequence information. Only two of the genetic maps relate to white jute and no QTL mapping has been reported [2, 20]. Thus, high-density genetic maps for white jute are lacking; in particular, a map that covers a large number of molecular markers with sufficient sequence information is needed to meet the demand for QTL mapping and other types of research.

In the last decade, many molecular marker technologies have been developed, including RAPD, AFLP, ISSR, SRAP, and SSR [14]. All of these DNA-based molecular markers can be used for linkage map construction. However, these markers are time-consuming and costly to prepare and some have proven to be unstable [22, 23]. Recent developments in sequencing technology have simplified and accelerated the discovery of sequence variants, enabling the development of sequence-based markers including SNPs and insertion/deletion polymorphism (InDel) markers [24]. SNPs are more useful as genetic markers because they are the most abundant and stable form of genetic variation in most genomes [25, 26]. They have been used for genetic linkage mapping in many organisms including soybean, barley, cabbage, oilseed rape, and sugar beet [9, 27–30]. Specific locus amplified fragment sequencing (SLAF-seq) has been proven to be an efficient method of large-scale *de novo* SNP discovery and genotyping using high-throughput sequencing, and it provides a high-resolution strategy for large-scale genotyping that is applicable to a wide range of species and populations [31]. The efficiency of this approach has been demonstrated in rice and soybean, and it has also been used to create a genetic map for common carp (*Cyprinus carpio* L.) without the benefit of a reference genome sequence [32].

In this study, we used SLAF-seq to develop SNP and InDel markers and then constructed a high-density genetic map for white jute. The characteristics of this map were investigated. Eleven QTLs associated with plant height were identified using this new genetic linkage map.

## Methods

### Mapping population and phenotyping

To develop an RIL mapping population of *C. capsularis*, a cross was made between elite cultivar ‘179’ (female parent) and local variety ‘Aidianyesheng’ (male parent, supplied by the National Medium-term Genebank of Jute Germplasm Resources). Cultivar ‘179’ was derived from the cross ‘Meifeng No. 2’ and ‘Minma No. 5’; ‘Aidianyesheng’ is a long-established local variety. The mapping population consisted of 100 individuals and was developed by single seed descent (F_8_) at Fujian Agriculture and Forestry University, Fuzhou and Hainan, China. The F_8_ families together with their two founders were grown at two different locations, namely, the experimental fields of Fujian Agriculture and Forestry University, Hongwei in 2011 (26.10°N, 119.01°E, 26 m above m.s.l.) and Yangzhong in 2012 (26.16°N, 118.28°E, 191 m above m.s.l.). At each location, the experiment was conducted from May to October using standard cultural practices; the plants were positioned using a randomized complete block design with three replicates. Each genotype was raised in a double row of 50 plants with a spacing of 50 cm within the rows and 100 cm spacing between the replicates. Five healthy plants were harvested from each replicate at 130 day after sowing. Plant height (m) was recorded as the length of the undivided stem from the base of the plant to the point of bifurcation at the top.

### DNA isolation

Leaves from the two parents and the F_8_ lines were collected from seedlings and used for DNA extraction. Total genomic DNA was prepared from each plant according to the modified cetyltrimethyl ammonium bromide (CTAB) method [33]. DNA concentration and quality were estimated with a JS-2012 spectrophotometer and by electrophoresis using 1.2% agarose gels.

### SLAF library generation and sequencing

The SLAF library was generated using the protocol described by Sun et al. [31] with slight modifications. In brief, genomic DNA of each sample was treated with MseI (NEB, Ipswich, MA, USA), T_4_ DNA ligase (NEB), ATP (NEB), and MseI adapter at 37 °C. Restriction/ligation reactions were heat-inactivated at 65 °C and digested with BfaI and EcoRI restriction enzymes at 37 °C. Then, polymerase chain reaction (PCR) amplification was carried out in the reaction solutions containing the diluted restriction/ligation samples, dNTP, Taq DNA polymerase (NEB), and MseI-primer containing barcode 1. The PCR products were purified by the E.Z.N.A. Cycle Pure Kit (Omega, London, UK). The purified PCR products were pooled and incubated at 37 °C with MseI, T_4_ DNA ligase, ATP, and Solexa adapter. After incubation, the reaction products were purified using a Quick Spin column (Qiagen, Venlo, Netherlands), and electrophoresed on a 2% agarose gel. SLAFs of 350–380 bp and 500–550 bp (including adapter sequence indexes and adaptors) were isolated using Gel Extraction Kits (Qiagen). These SLAFs were subjected to PCR with a Phusion Master Mix (NEB) and Solexa amplification primer mix to add barcode 2. PCR products were gel purified and SLAFs of 280–310 bp and 430–480 bp were selected for paired-end sequencing on an Illumina HiSeq 2500 sequencing platform (Illumina, San Diego, CA, USA). According to the barcode sequences, raw reads were demultiplexed to individual reads. Then, low quality reads (quality score <20) were filtered out and the good quality reads were used for molecular marker discovery.

### SLAF-seq data grouping and genotyping

All SLAF pair-end reads with clear index information were clustered using sequence similarity as detected by BLAT [34] (−tilesize = 10 -stepsize = 5). All SLAF markers were filtered four times and quality was assessed by the method described by Sun et al. [31]. Sequences that clustered together were defined as an SLAF locus. All polymorphic SLAF loci were genotyped for consistency in parents and progenies. Jute is a diploid species; therefore, one locus will contain a maximum of four SLAF tags. Groups containing more than four tags were filtered out as repetitive SLAFs. In this study, SLAFs with a sequence depth of less than 10 were defined as low-depth SLAFs and were filtered out. A SLAF which had less than three SNPs and average depth of each sample above three, was used as a high quality SLAF marker. Polymorphic markers were classified into eight segregation patterns (aa × bb, ab × cc, ab × cd, cc × ab, ef × eg, hk × hk, lm × ll and nn × np). An F_8_ population is obtained from a cross of two diverse parents with the genotype aa or bb. Therefore, our study only used SLAF markers with the segregation pattern aa × bb for genetic map construction. The cut-off value for the proportion of missing data is 30%.

### Linkage map construction

In this study, we performed a 2-point linkage analysis using efficient SLAFs after genotyping the 100 RILs. A high-density genetic map including 11 LGs was constructed using the grouping function of Joinmap v4.0 software [35]. Modified logarithm of odds (MLOD) scores between markers were calculated to confirm the robustness of the markers for each LG. Markers with MLOD scores <5 were filtered prior to ordering. A LOD threshold of 3.0 was set as the default. High Map Strategy was used to order SLAF markers and correct genotyping errors within the LGs [36]. The LGs were then analyzed as described by Zhang et al. [37]: primary marker order was established by their location on chromosomes, according to the relationships between ordered markers; genotyping errors or deletions were corrected using the SMOOTH algorithm; MST map was used to order the map; the new ordered genotypes were corrected with the SMOOTH algorithm; and the Kosambi mapping function was used to estimate map distances [38].

### Plant height evaluation and QTL mapping analysis

The frequency distribution of plant heights in the two growing years was analyzed. A QTL analysis was then performed using the mean data and the Ici Mapping package [39]. Plant height QTLs were identified by internal mapping methods with the package R/QTL [40]. LOD significance thresholds for QTL peaks were determined using 1,000 permutations. Results from the interval mapping analysis were used to construct the QTLs, and their positions were used in a default model. Other parameters were set as follows: the step for scanning was 1.0 cM, since many adjacent markers in the SNP linkage map had a map distance of <1 cM; and the largest *P*-value for entering variables in stepwise regression of phenotype on marker variables (PIN) was 0.001. The percentage of variance explained and the additive effect were estimated for each QTL.

## Results

### SLAF sequencing and genotyping

**Fig. 1:**
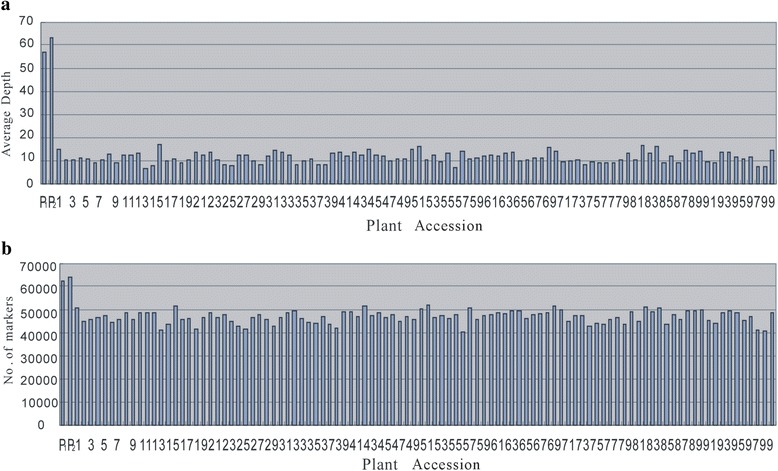
Depth and number of markers for each of the F_8_ individual and their parents. The *x*-axis in **a** and **b** indicates the plant accession including the female parent and the male parent followed by each of the F_8_ individuals, the y-axis indicates depth in a and number of markers in b

**Fig. 2:**
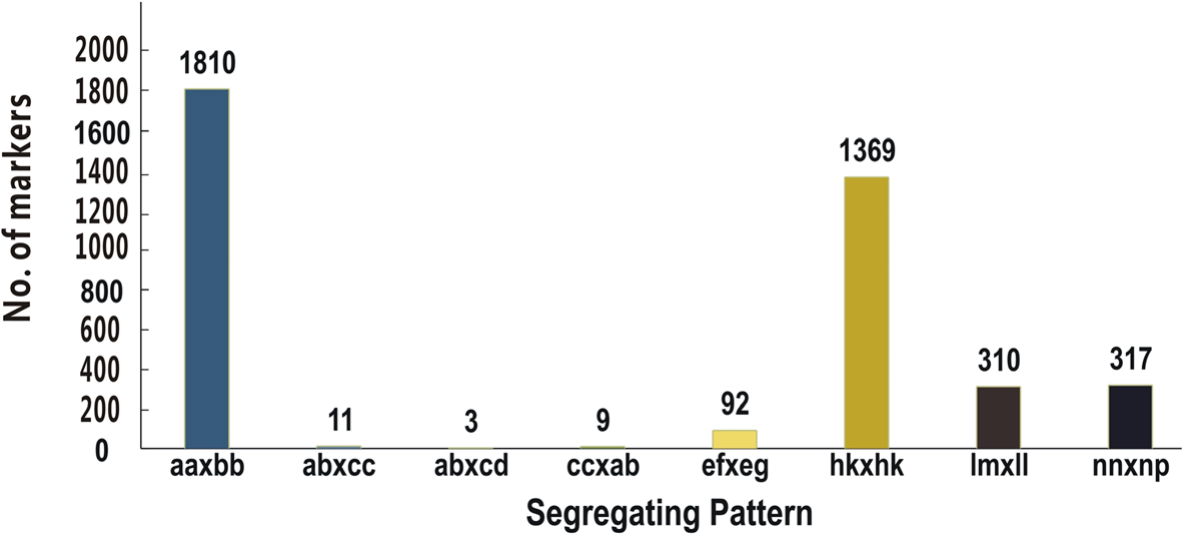
Number of markers for eight segregation patterns

DNA sequencing generated 43.88 Gb of raw data consisting of 135,195,254 pair-end reads, each of about 90 bp after preprocessing. The high-quality base rate was 78.23%, and guanine-cytosine (GC) content was 41.11%, with quality scores of at least Q20 (Q20, indicating a 1% chance of an error, and thus 99% confidence). In total, 69,446 SLAFs were detected, with an average sequencing depth of 56.81 in ‘179’, 62.91 in ‘Aidianyesheng’, and 11.49 in the progeny (Fig. 1). Of these SLAFs, 5,074 (7.31%) were polymorphic (Table 1). After filtering out SLAFs lacking parental information, 3,921 remained and these were classified into eight segregation patterns (Fig. 2). After filtering out low quality SLAF markers and heterozygous parental markers, 913 markers with high quality were used to construct a linkage map. The average sequencing depths of these markers were 43.11-fold in ‘179’, 43.33-fold in ‘Aidianyesheng’, and 9.83-fold in RIL individuals.

**Table 1:** SLAF markers mining results

Type of markers	Number of SLAF markers	Total depth	Ratio
Polymorphisms	5,074	3,124,714	7.31%
Non-polymorphisms	64,372	42,268,156	92.69%
Total	69,446	45,392,870	100%

### High-density linkage map construction

**Fig. 3:**
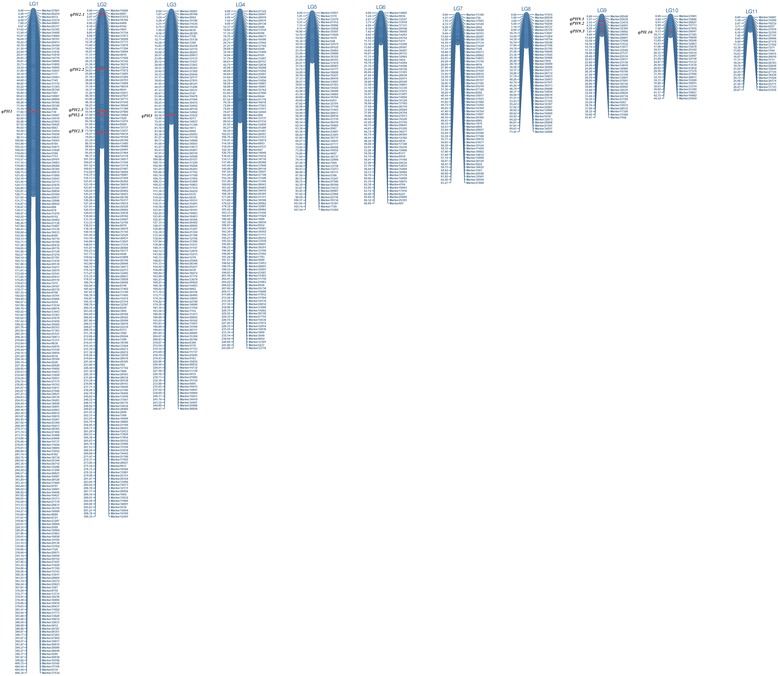
High-density linkage map and QTLs associated with plant height for white jute

The 913 markers were assigned to 11 LGs, including 7 major LGs and 4 minor LGs. The map spanned a total of 1621.42 cM with an average distance of 1.61 cM between adjacent markers (Table 2, Fig. 3). As shown in Table 2, the largest LG was LG1 with 210 markers, a total length of 406.34 cM, and an average distance of 1.93 cM between adjacent markers. The smallest LG was LG11, with only 25 markers and a length of 29.66 cM. The degree of linkage between markers was reflected by a gap ≤5 ranging from 99.04 to 100% with an average value of 99.91%. The largest gap on the map was 5.18 cM located in LG1.

**Table 2:** Description on basic characteristics of the 11 LGs

Linkage group ID	Marker number	Total distance (cM)	Average distance (cM)	Largest Gap	Gaps < = 5
LG1	210	406.34	1.93	5.18	99.04%
LG2	161	300.35	1.87	4.16	100%
LG3	127	246.46	1.94	4.16	100%
LG4	108	243.60	2.26	4.42	100%
LG5	64	107.53	1.68	3.77	100%
LG6	62	66.99	1.08	2.76	100%
LG7	55	63.26	1.15	2.8	100%
LG8	39	71.41	1.83	2.82	100%
LG9	34	41.47	1.22	1.57	100%
LG10	28	44.31	1.58	2.84	100%
LG11	25	29.66	1.19	1.24	100%
Max linkage group	210	406.34	1.93	5.18	99.04%
Min linkage group	25	29.66	1.19	1.24	100%
Total	913	1621.42			
Average	83.00	147.40	1.61	3.25	99.91%

‘Gap < =5’ indicates the percentages of gaps in which the distance between adjacent markers was smaller than 5 cM

### Distribution of SLAF markers on the genetic map

**Fig. 4:**
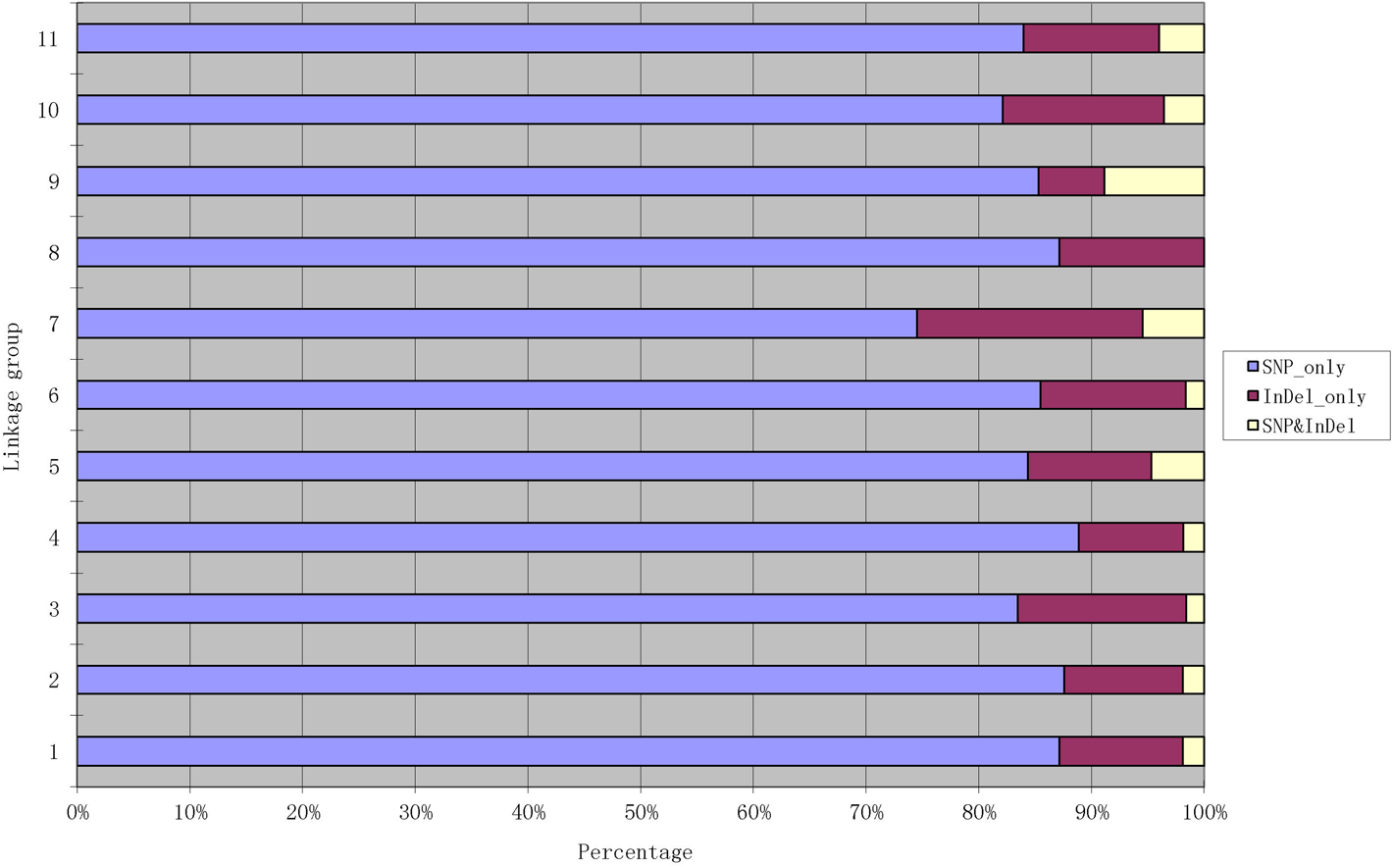
Percentages of diverse types of markers on each linkage group

The 913 markers included 781 ‘SNP_only,’ 109 ‘InDel_only,’ and 23 ‘SNP&InDel’ markers. The distribution of each of these markers on the genetic map and in each LG was investigated (Table 3, Fig. 4). ‘SNP_only’ accounted for 85.54% of the markers and was the predominant type. The proportions of the three types of markers on LG1 were 87.14%, 10.95, and 1.90%, respectively, which was similar to the average proportions of marker types for all 11 LGs. LG7 had the lowest proportion of ‘SNP_only’ markers but had the highest rate of ‘InDel_only’ markers at 74.55 and 20% respectively. LG4 had the highest proportion of ‘SNP_only’ markers but had the lowest rate of ‘InDel_only’ at 88.89 and 9.25%.

**Table 3:** Description on type of the markers

Linkage group	Number of total markers	SNP_only	InDel_ only	SNP&InDel
1	210	183	23	4
2	161	141	17	3
3	127	106	19	2
4	108	96	10	2
5	64	54	7	3
6	62	53	8	1
7	55	41	11	3
8	39	34	5	0
9	34	29	2	3
10	28	23	4	1
11	25	21	3	1
Total	913	781	109	23
Average	83	71	10	2

The nature of the 781 SNP loci was investigated (Table 4). Most were transition type SNPs of R (G/A) and Y (T/C) types with rates of 34.11% and 34.96%, respectively. Four transversion type SNPs were identified including K (G/T), M (A/C), S (G/C) and W (A/T) with frequencies ranging from 5.95 to 9.25%; these types of SNP accounted for 30.93% of all SNPs. These results are very similar to those reported in sesame [31].

**Table 4:** Statistic of mapped SNP marker types

Type	Number	Ratio
M(A/C)	66	8.40%
R(A/G)	266	34.11%
Y(C/T)	273	34.96%
S(C/G)	46	5.95%
W(A/T)	72	9.25%
K(G/T)	57	7.33%
Total	781	100%

### Segregation distortion markers

Analysis of the segregation of the 913 mapped loci showed that 862 (94.4%) deviated significantly (*P* ≤ 0.05) from the expected 1:1 Mendelian segregation ratio. The result showed that segregation distortion markers were present on every LG (Table 5). LG2 had the highest proportion of segregation distortion markers at 95.03%, and LG7 had the lowest rate at 83.64%.

**Table 5:** Distribution of segregation distortion markers

Linkage group	Number of total markers	Percentage	Number of segregation distortion markers	Percentage
1	210	23.00%	184	87.62%
2	161	17.63%	153	95.03%
3	127	13.91%	115	90.55%
4	108	11.83%	102	94.44%
5	64	7.01%	59	92.19%
6	62	6.79%	56	90.32%
7	55	6.02%	46	83.64%
8	39	4.27%	36	92.31%
9	34	3.72%	30	88.24%
10	28	3.07%	26	92.86%
11	25	2.74%	23	92.00%
Total	913			

### QTL mapping of plant height

#### Frequency distribution for plant height

**Fig. 5:**
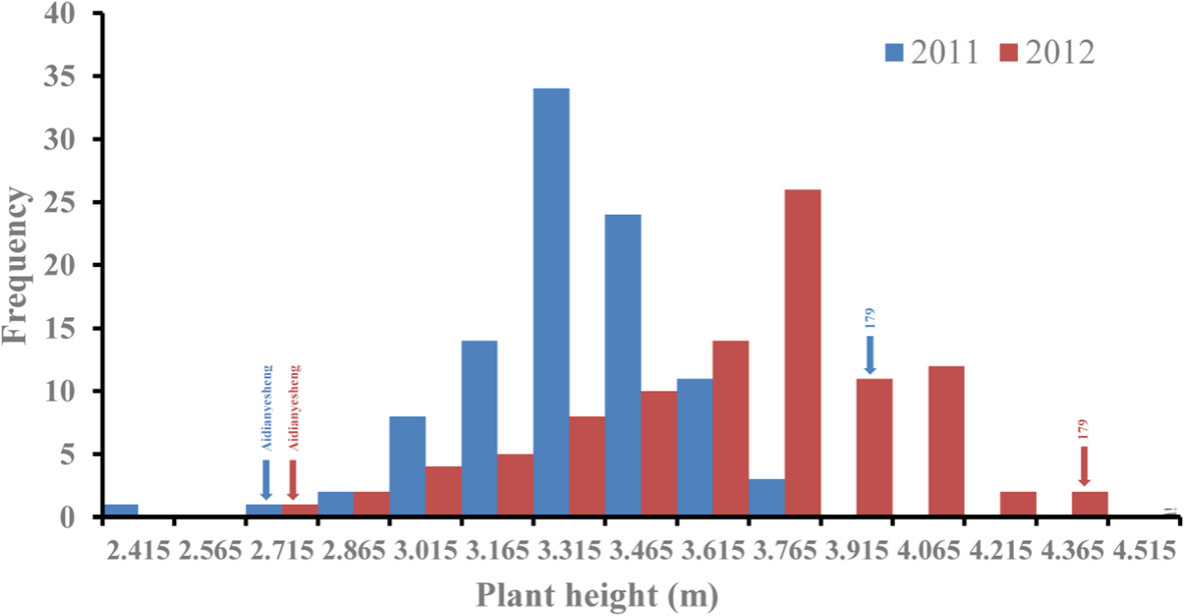
Frequency distribution of plant height in the RIL population of white jute in 2011 and 2012. The *x*-axis indicates the plant height of the RIL population and the parents, and the y-axis indicates the distributing frequency of plant height. Arrows indicate the distributing frequency of plant height for each parent

A comparison of the parental cultivars showed significant differences in plant height (Fig. 5). Average plant height in ‘ 179’ was 3.93 m, which was significantly greater than in ‘Aidianyesheng’ (2.64 m) in 2011, and a similar difference was also found in 2012. Plant heights in the RIL population showed continuous variation (Fig. 5). A comparison of the results from the two locations showed that plant heights were significantly influenced by the environment.

### QTL mapping in white jute

Eleven stable QTLs for plant height were identified using the SLAF linkage map across the two locations and the pooled data (Table 6); these QTLs explained 4.14 –15.63% of phenotypic variance. The QTLs mapped to 5 LGs named LG1, LG2, LG3, LG9, and LG10 (Table 6 and Fig. 3). Five plant height QTLs were detected on LG2, three were mapped on LG9, and one each on LG1, LG3, and LG10. The plant height QTL located at 223.0 cM on LG2 (between markers 595 and 17742) was found to have a major effect and accounted 15.63% of the phenotypic variance with a LOD score of 7.20. Most of the additive effects were negative for QTLs of plant height, indicating that increased trait values were conferred by the female (‘179’) alleles.

**Table 6:** QTLs for plant height in the RIL population in both 2011 and 2012

QTL	Position(cM)	Left Marker	Right Marker	LOD	PVE	ADD
*qPH1*	222.5–225.5	Marker4534	Marker36189	2.65	4.14	−0.05
*qPH2.1*	8.5–10.5	Marker31754	Marker37873	4.93	9.37	0.07
*qPH2.2*	129.5–131.5	Marker29182	Marker24007	4.81	9.51	0.07
*qPH2.3*	222.5–225.5	Marker595	Marker17742	7.20	15.63	−0.08
*qPH2.4*	230.5–233.5	Marker28534	Marker26538	3.16	5.41	−0.04
*qPH2.5*	269.5–270.5	Marker34442	Marker21780	5.27	9.91	−0.08
*qPH3*	229.5–231.5	Marker21995	Marker35742	3.33	6.33	−0.06
*qPH9.1*	9.5–12.5	Marker29916	Marker20881	2.84	4.73	−0.06
*qPH9.2*	17.5–21.5	Marker27537	Marker25534	3.47	5.55	−0.06
*qPH9.3*	30.5–36.5	Marker5567	Marker15972	3.22	5.35	−0.06
*qPH10*	33.5–37.5	Marker16051	Marker33314	2.92	4.84	−0.06

## Discussion

### The need for development of genetic markers for white jute

Previous studies used traditional markers and newly developed markers to construct genetic linkage maps in jute [17, 18, 20]; however, only a few studies were conducted in white jute. Although genetic maps for jute exist, the total number of markers on the LGs is limited [16–19], and do not provide comprehensive coverage of the jute genome. In general, a high number of polymorphic markers is necessary to guarantee the accuracy of a linkage map [2]. The limited number of available markers and their low polymorphism rate made construction of a genetic linkage map with high-density markers for white jute almost impossible. Other markers, such as SSRs and SNPs, clearly need to be developed to overcome the scarcity of DNA markers for construction of saturated genetic maps in jute [41, 42]. Biswas et al. constructed a linkage map in white jute using SNP markers that were based on expressed sequence tags and not sequencing [21]. Genotyping by sequencing is a high-throughput technique for the efficient development of large numbers of markers in a short time to generate polymorphic markers for high-density genetic map construction. In our study, a high density genetic map of white jute was constructed using SLAF-seq genotyping data. The constructed genetic map had a higher density of the maps made of organism lacking reference genome sequences.

### SLAF-seq is an ideal approach for developing markers

The SLAF-seq strategy is better than other sequencing approaches because it combines locus-specific amplification and high-throughput sequencing technology, although reference genome sequences and polymorphism information are not necessary to use this strategy [31]. In contrast to conventional methods, which are inefficient, expensive, and time-consuming, [43, 44], SLAF sequencing can generate large amounts of sequence information and handle whole genome density distributions, which ensures density, uniformity, and efficiency of marker development. Since SLAF-seq methods were first developed, they have been used in map construction for sesame, soybean, oil rape, and other vegetable crops [7, 11]. Our study provides the first development of markers on a large scale for white jute. In total, 69,446 SLAF markers were developed using high throughput sequencing, and 5,074 of these were polymorphic. We obtained 913 markers that were suitable for constructing a linkage map. Marker integrity and accuracy were high and marker quality and quantity met the requirements for construction of a genetic map. Therefore, the SLAF-seq technology is ideal for developing plant chromosome-specific molecular markers with high success rates, specificity, and stability.

### Inconsistency between LGs and chromosomes

To our knowledge, the genetic map presented in this paper is the densest map to date for white jute. However, our map is still not saturated, because it has 11 LGs and not 7 LGs as was expected. Several factors may be responsible, such as the genetic constitution of different mapping populations, mapping strategies, number and type of mapped loci, the choice of mapping software, and ratio between number of markers and population size [20]. In the present study, an unexpectedly high level of heterozygosity in the two parents resulted in some markers that could not be used for linkage analysis, and this may have resulted in larger gaps between adjacent markers. Additionally, if the markers were unevenly distributed on the map, an increased number of LGs might have been produced. When compared with previous studies in jute [15, 20, 21], the size of the populations used in this study was relatively low, which may have contributed to the inconsistency in numbers of LGs and chromosomes. In addition, the marked markers are clearly not sufficiently abundant. The fairly low genetic diversity at the species level in jute [15] may be a major reason for the low proportion of polymorphic SLAF markers in the present study. We assumed some fragments of chromosomes in two parents have same genetic background, so no polymorphic markers were detected in these sections. The fragments which should be jointed in the same LG may have been separated into different LGs because of this reason. Although we attempted to develop and add more polymorphic markers, the unexpectedly low polymorphism information content in combination with an inherently narrow genetic base makes the reduction in the jute LG number impossible at present.

### Marker segregation distortion

In our study, an unusually high degree of marker segregation distortion was observed. The rate was higher than those reported by Das et al. [20] and Topdar et al. [16]. Segregation distortion is widespread in inter-specific and intra-specific crosses used to construct mapping populations [45]. Distorting factors can be self-incompatibility alleles, deleterious recessive alleles, structural rearrangements, or differences in DNA content [46]. High levels of segregation distortion might also be the result of use of genetically distant parents [47]. Of the two parental lines used in this study, ‘Aidianyesheng’ is a long-established local line while ‘179’ is a cultivated accession; the differences in their traits indicate they are genotypically divergent and that there is a high degree of genetic variation between them. This may account for the high level of segregation distortion in this study. Semagn et al. also reported high segregation distortion in doubled haploid (DH) and RIL populations [47]. In Arabidopsis, a high level of segregation distortion was attributed to a higher frequency of recombination [48]. Nevertheless, segregation distortion has very little effect on marker order and map length [49]. Zhang et al. [50] showed that segregation distortion could result in higher genetic variance than non-distortion and help to improve the detection of linked QTLs, because distortion markers do not have a large effect on the position or effect estimations of QTL analysis.

### High-density genetic map and QTL mapping

Our map covers 1,621.42 cM with an average of 83 markers per LG and an average distance of 1.61 cM between adjacent markers. This is the smallest average inter-marker distance reported for white jute. Furthermore, the map constructed in this study has the largest number of markers for jute. More importantly, 85.54% of the markers on this genetic map were SNPs, i.e., sequence tagged markers with co-dominant inheritance, that are suitable for comparative genomic studies [51] and association mapping [52, 53]. The high resolution genetic map developed here will be a useful platform for the assembly of the white jute genome, for the development of sequence-based markers used in breeding programs, and for the identification of genes associated with important agricultural traits.

During the present study, 11 QTLs associated with plant height, including one major effect QTL, were identified on LG1, LG2, LG3, LG9, and LG10. The QTLs detected in this study were stable in the two cultivation locations. The selection of parents can affect the accuracy and utility of QTL mapping. In the present study, plant height in ‘179’ (female parent) was significantly greater than in ‘Aidianyesheng’ (male parent). We found the female parent contributed the main QTL increasing plant height. Topdar et al. found increased plant height was conferred by the female (taller) allele at three loci and the male (shorter) allele at only one locus [16]. Kundu et al. showed that enhancing alleles of two plant height QTLs were from male and female parent respectively, and they also found plant height was strongly correlated with fiber yield [15].

As we used a different species of jute here compared to previous reports, we could not combine and compare the identified QTLs for plant height among studies [15, 16]. It is unclear whether these QTLs were in the same location or on the same LG as found in previous studies. We expect that further research on QTLs associated with yield traits will improve the breeding efficiency of jute. The molecular markers linked with fiber yield such as plant height may be used in marker assisted selection to accelerate development of cultivars with high fiber yield and quality.

## Conclusions

In this study, a high density genetic map of white jute was constructed using SLAF-seq technique. To our knowledge, this map is the densest map to date for white jute. Furthermore, 11 QTLs associated with plant height, including one major effect QTL, were identified. The results will provide an important platform for further development in both molecular biology and breeding of white jute.

## Abbreviations

CTAB: Cetyltrimethy l ammonium bromide
DH: Doubled haploid
GC: Guanine-cytosine
InDel: Insertion/deletion Polymorphism
ISSR: Inter-simple sequence repeats
LG: Linkage group
MLOD: Modified logarithm of odds
QTL: Quantitative trait locus
RAD: Restriction Site Associated DNA
RAPD: Randomly amplified polymorphic DNA
RIL: Recombinant inbred lines
SLAF-seq: Specific locus amplified fragment sequencing
SNP: Single nucleotide polymorphism
SRAP﻿: Sequence-related amplified polymorphism﻿
SSR: Simple sequence repeats
